# Using Objective Structured Teaching Encounters (OSTEs) to prepare chief residents to be emotionally intelligent leaders

**DOI:** 10.1080/10872981.2017.1320186

**Published:** 2017-05-02

**Authors:** Sara Ann Cerrone, Patti Adelman, Salaahuddin Akbar, Andrew C. Yacht, Alice Fornari

**Affiliations:** ^a^ Internal Medicine, Northwell Health, Manhasset, NY, USA; ^b^ Center for Learn and Innovation & Physician Leadership Institute, Lake Success, NY, USA; ^c^ Learning and Organizational Effectiveness, Northwell Health, Great Neck, NY, USA; ^d^ Office of Academic Affairs, Northwell Health, Great Neck, NY, USA; ^e^ Faculty Development, Northwell Health, Great Neck, NY, USA; ^f^ Educational Skills Development, Hofstra Northwell School of Medicine, Manhasset, NY, USA; ^g^ Science Education, Population Health and Family Medicine, Northwell Health, Great Neck, NY, USA

**Keywords:** Leadership training, chief residents, graduate medical education, OSTEs, emotional intelligence

## Abstract

**Background**: Chief Residents must lead, manage and mentor a diverse and often large group of residents, however there is a lack of formal leadership training throughout graduate medical education.

**Objective**: Development of a 3-part Chief Resident (CR) Program focused on leading, managing and mentoring.

**Design:** Each participant completes an Emotional Intelligence (EI) Inventory prior to the day-long event. Participants receive their EI scores at the beginning of the program, which features interactive sessions on leadership, management, and feedback skills. The program then reinforces the application of their new knowledge about EI through a four station OSTE (Observed Structured Teaching Encounter). CRs practice feedback and coaching skills in a simulated environment where they need to provide the context of formative feedback to a standardized resident.

**Results**: The aggregated mean pre-session EI score for all participants was 76.9 (an ideal score is >85). An independent-samples t-test compared the CRs’ leadership and feedback performance on their first and second OSTE performance within a single afternoon session. There was a significant difference between the first OSTE performance (M = 47.92, SD = 7.8) and the second OSTE performance (M = 51.22, SD = 6.9); t (68) = 1.99, p = 0.006. These results suggest that participating in multiple OSTEs positively reinforces the core interpersonal and communication skills discussed in the didactic and practiced in the interactive portions of the program.

**Conclusion**: The low mean pre-session EI score achieved by our participants supports the idea that CRs enter their new roles with a level of EI that can be enhanced. CRs had an overall positive reaction to EI and its application to the core skills addressed in the program, highlighting the fact that similar programs could be used to train early career physicians to be more skilled and comfortable with leading, managing and mentoring.

**Abbreviations**: CR: Chief resident; EI: Emotional intelligence; GME: Graduate medical education; OSTE: Objective structured teaching encounter

## Introduction

Chief Residents (CRs) are typically chosen based on their clinical work as residents or as a set role during the final year of residency. Described as early as the 19th century, chief residents refer to senior residents that have demonstrated competence to manage and care for patients with minimal supervision [1]. This role has evolved over the past century and the responsibilities of CRs vary among specialties, but what has remained constant is that CRs all are required to lead and mentor a diverse and often large group of residents. CRs tend to be responsible for multiple administrative, educational, and clinical components throughout the year. CRs oversee resident scheduling, compliance of programs with credentialing councils, as well as being supervisors of resident education [1]. In addition to these administrative responsibilities, CRs learn how to be leaders by acting as a liaison between junior residents and clinical program leadership, resolving resident and interdisciplinary conflicts, and navigating the healthcare environment and health systems that involve senior directors and administrators. Effective clinician leadership has been shown to improve patient care by encouraging teamwork, promoting a culture that supports safe practices, and enabling innovation and continuous development of skills to promote better outcomes for patients and health-care systems [2]. Being an effective leader is vital for success as a CR, though, CRs are not often chosen based on their ability to lead. In medical school and the early years of internship and residency, individuals focus on their clinical growth and expertise and have minimal exposure to leadership skills development [1,3]. One such explanation for a lack of formal leadership training is that the ‘creativity, innovation, and strategic insight required for successful leadership and management’ are often seen as being at odds with the analytical skills that are the focus of medical education [3]. Despite this lack of training, CRs are expected to demonstrate specific leadership skills to meet the demands of their newly acquired and influential roles.

Gewertz and Logan state that the challenge facing physicians, including CRs, who ‘aspire to outstanding leadership is to continue to ’own‘ what makes them star performers as individuals, while shifting their focus to others’ [4]. As CRs transition into their new leadership roles, they must display a dual set of knowledge and expertise; an understanding of clinical medicine and additional proficiency in traditional business content [3]. CRs, while maintaining a clinical leadership presence, must also transition to a new set of rules; focusing more on how their team’s work gets done and less on the results themselves [4]. This is not to say that clinical results no longer matter, but in leadership, there is a recognition that ‘how results are achieved matters as much, and often much more.’ [4]

One of the skills found to be central to leadership, and can therefore ensure a smooth transition, is Emotional Intelligence (EI). EI is a relatively recent concept to be embraced within the organizational sphere; however, its definition can be dated back to the early 1900s [5]. EI is associated with leadership success and encompasses four competencies: self-awareness, self-management, social awareness, and relationship management [5]. The four-component model includes two distinct but interconnected domains; personal competence and social competence. To enact correctly, these four elements require the coordination of both the rational (thinking) mind and the emotional (feeling) mind. Daniel Goleman posits that while these two minds are well coordinated the majority of time and ‘feelings are essential to thought, [and] thought to feeling’, he recognizes that when the balance tips, it is often the emotional mind that captures the upper hand [5]. Through EI teaching, an individual learns to first recognize his or her emotional response to a situation and to then manage this emotional response internally in an effort to improve his or her efficiency with decision making skills and positively influence outcomes [2]. Substantial evidence in multiple professional fields shows that EI measures abilities critical for leaders and correlates with high performance. At the very highest levels, competence models for leadership typically range from 80 to 100 percent EI-related abilities [5]. Many medical communities are acknowledging the value of EI skills for physician development as it promotes patient safety and satisfaction in addition to improving inter-professional cooperation [6,7]. EI has been suggested for physician trainees for teaching interpersonal skills, communication, and professionalism, all of which contribute to the core competencies of the Accreditation Council for Graduate Medical Education (ACGME) [8]. Various studies have also shown that medical professionals want EI training, yet there is a gap between the recognition of the need for EI training and the development of EI curricula in graduate medical education programs, with very little research focused on how EI can best be learned and assessed [2,4,9]. Furthermore, there is no known average or accepted emotional intelligence score among resident physicians and that any EI intervention must be tailored to an individual resident or particular resident group [10]. Leadership training courses have been implemented in various graduate medical education programs and some have focused on CR development through lecture series or formal mentorship programs [6,11–13]. Analysis of the benefit of these programs is based on post-program self-assessment surveys filled out by participants, but there does not seem to be a standard or optimal format for these programs that provides the most benefit.

The use of standardized learners in Objective Structured Teaching Encounters (OSTEs) has proven to be effective in assessing teaching skills of medical school faculty, residents, and medical students [14–16]. OSTEs are a pedagogy that support active application of learning after the delivery of content and function to assure actual learning is occurring. OSTEs also provide the framework to evaluate specific teaching competences with direct feedback given at the end of each encounter [15–18]. Most leadership development programs for physicians minimally use interactive learning and feedback [16,17]. OSTEs, though, have been utilized to provide faculty development and feedback performance within the realm of undergraduate and graduate medical education [14,15]. Given that OSTEs have been successful in these areas, it is reasonable to consider their use for leadership development and training. We believe that through this type of learning-in-action, with attention paid to emotional intelligence that new leadership and feedback skills can be expediently practiced to assure CRs are better prepared for their responsibilities as educational leaders.

**Figure 1.**
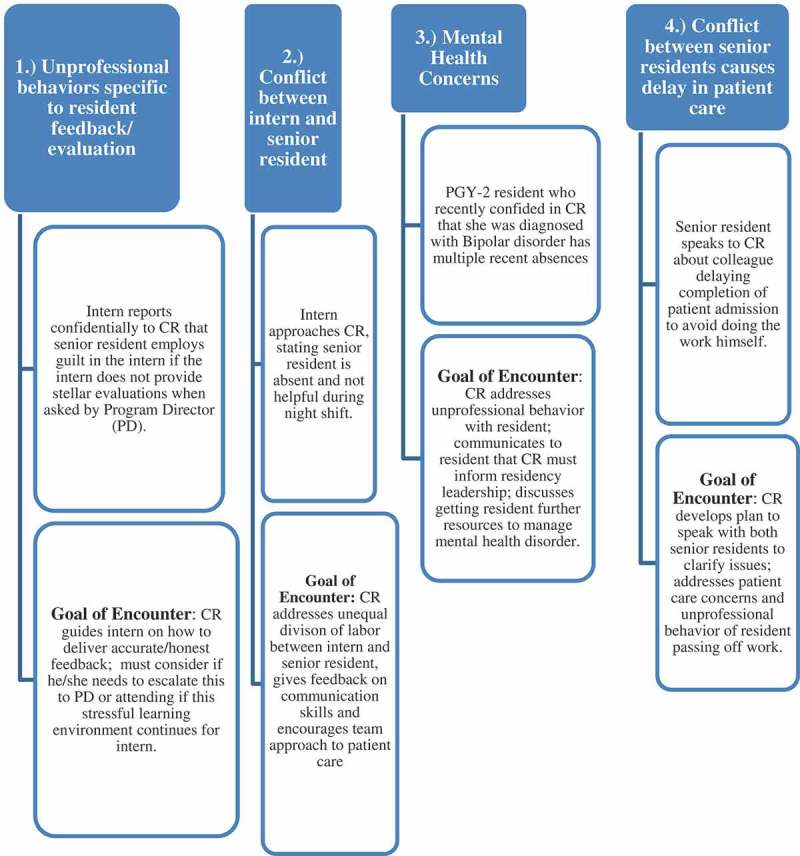
Sample OSTE content.

Our program began with each participant completing an EI Inventory three weeks prior to the day-long program. The daylong experience begins with a series of didactic sessions that have a focus on leadership, managing, and core feedback skills. During this portion of the course, participants received their individual EI report, interpretation of their scores, and strategies to improve them. The third part of the program, which takes place during the second half of the day, reinforces the application of new knowledge through a four station OSTE skill building experience (Figure 1). This highly experiential portion of the course focuses on leadership and mentoring specific to interpersonal and communication skills and professionalism, core competencies required by the ACGME accreditation system and all requiring the underpinnings of EI.

## Methods

Northwell Health is a large integrated health care organization consisting of 21 hospitals and over 450 ambulatory and research sites. Northwell Health is the institutional sponsor of 120 accredited and non-accredited GME training programs, with approximately 1600 residents and 100 chief residents annually. Incoming CRs from multiple GME programs (N = 100, 15 disciplines) were recruited to participate in one of four one-day, nine-hour courses prior to the start of their CR year. Participant recruitment was achieved through advertising sent to program directors, coordinators, and current chief residents. We had a goal of 20–25 participants at each of the four sessions.

Participants completed an online version of the TalentSmart® EI inventory assessment, that was available through our health system, approximately three weeks prior to the course. Theinventory follows the revised Goleman model of emotional intelligence [5], which evaluates four separate components: self-awareness, self-management, social awareness and relationship management. The ‘consultant education’ version of this assessment was used and participants did not learn their scores until they attended the course. This prevented participants from focusing on the numbered result without first understanding the context of the score and the practical application to their future roles requiring new skills. On the day of the course, the results of the EI inventory were confidentially reviewed with each participant to clarify what the scores meant in order to identify existing strengths and areas for improvement. The intent was this new knowledge would be applied in the formative OSTE stations.

Senior training program educators (Program Directors, Leaders of Faculty Development and Physician Leadership Institute) lead sessions emphasizing leadership responsibilities applicable to the role of a CR, with a focus on skills necessary to deliver difficult feedback and strategies to assist with interpersonal conflict resolution among resident teams. The didactic session ended with a panel of current CRs joining the group to share their year-long experiences, thoughts on lessons learned as a CR, and best practices in leadership, managing, and mentoring.

**Figure 2.**
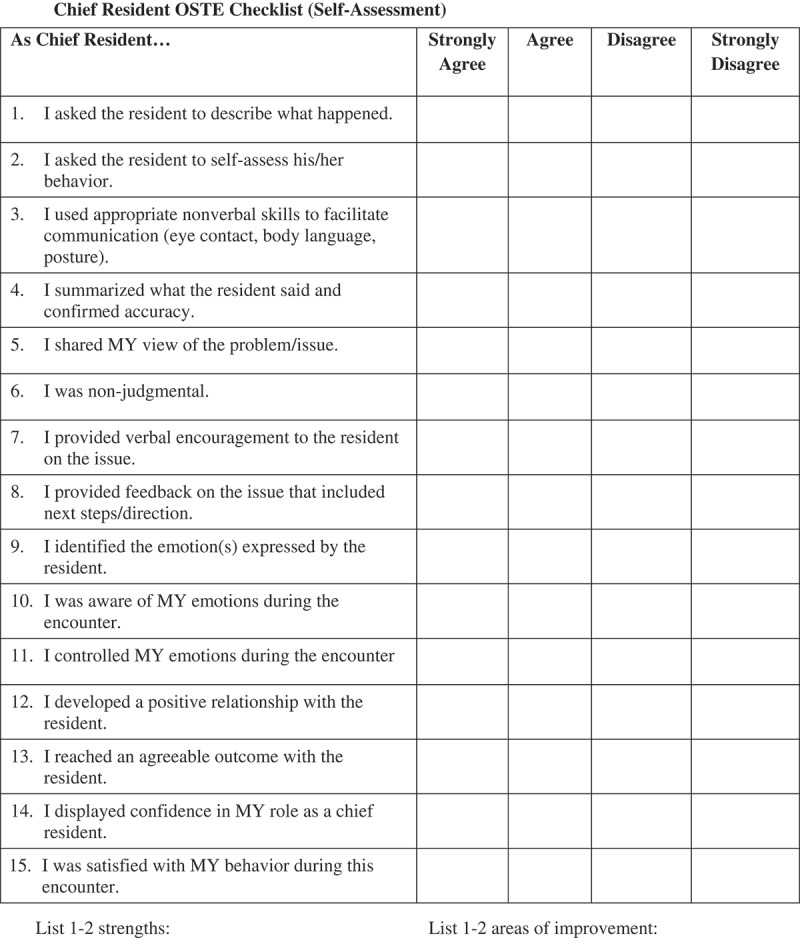
Sample feedback check list (self-assessment).

**Figure 3.**
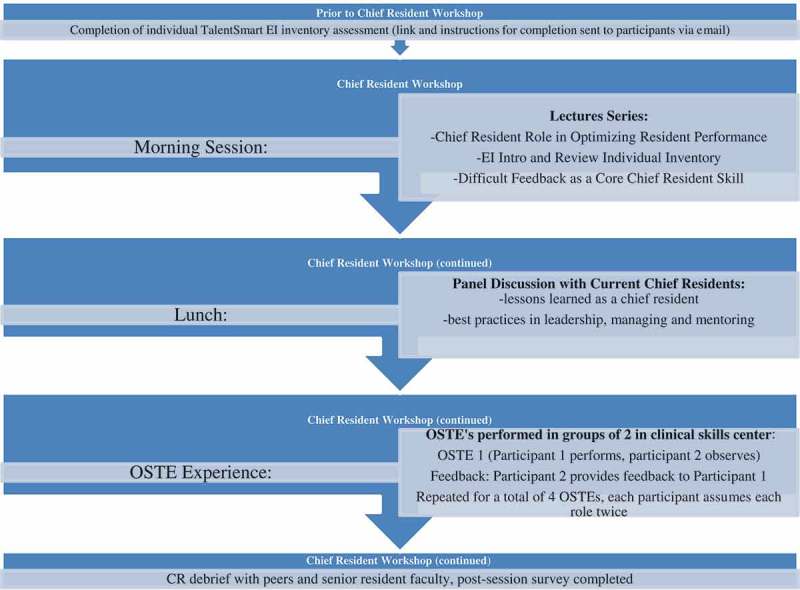
Outline of workshop.

The CRs were then divided into pairs to participate in OSTEs based on de-identified authentic incidents reported by previous CRs and collected anonymously using an electronic survey. In an effort to allow for more honest critique and feedback, the pairs purposely came from two different residency programs and individuals most often did not know their partners. The OSTE component occurred in a clinical skills center consisting of fourteen separate clinical encounter rooms. The standardized case script simulated clinical education situations in which the CRs needed to rely on EI to interact with and provide collaborative feedback to junior residents who presented difficult interpersonal and professionalism situations. Script content specifically addressed professionalism, confidentiality, issues of escalation, and teamwork (Figure 1). Actors in a standard format were educated to play the role of junior residents struggling with the several different scripted difficult situations. The CR pairs had two roles in the OSTE. One was to assume the role of the CR providing feedback and use EI skills with a junior resident presenting a difficult situation. The other role was to be a peer resident giving feedback to the CR learner. This was possible as the peer could observe and listen to the CR-resident interaction outside the encounter room. As there are four cases, each resident assumes each role twice. Feedback was organized via a checklist developed specifically to account for the skills addressed in the didactic sessions preceding the OSTEs. The CR, standardized resident, and peer observer each completed similar checklists prior to providing in person feedback (Figure 2). The encounter concluded with three-way feedback between the CR, standardized resident learner (actor), and peer resident observer who collectively reviewed the checklist to guide principles of collaborative coaching. The CRs debriefed as a group with their peers and a senior faculty member regarding communication, and professionalism behaviors, after the first two cases and then again after the last two cases. Debrief discussions focused on the content of the four OSTEs and making difficult decisions, maintaining confidentiality, and when to escalate issues or concerns to residency leadership. The debrief faculty completed a post session survey to document core issues addressed in the debrief session. A program agenda for the day is in Figure 3.

## Results

The program had 20 chief residents each day, to total 80 residents across 15 clinical disciplines and included all health system hospitals that had GME training programs.

The aggregated mean pre-session EI score for all participants was 76.9. The EI score can range from 0–100, and research has shown that strong leaders have a mean EI score of 85 or higher; less than a score of 50 is considered very undesirable [5]. The goal for our CR learners would be to move from their baseline score over time and progress to a higher score (85–100) as they continue their professional development as a physician.

**Figure 4.**
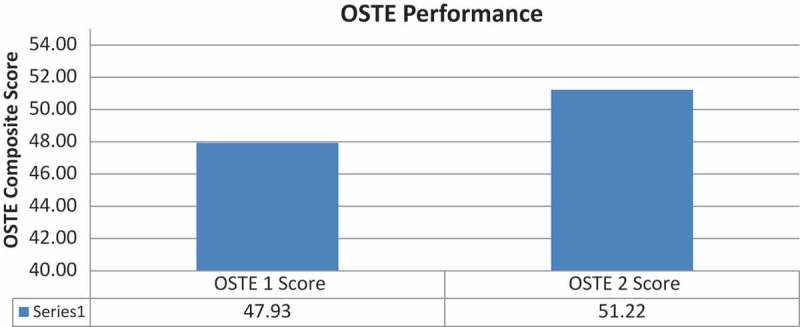
OSTE performance.

An independent-samples t-test was conducted to compare the CRs’ leadership and feedback performance as assessed by the CR, standardized learner and observer on the first and second OSTE performance. Performances were scored on a scale with a range of 0 to 100. There was a significant difference between the first OSTE performance (M = 47.92, SD = 7.8) and the second OSTE performance (M = 51.22, SD = 6.9); t (68) = 1.99, p = 0.006 (Figure 4). These results suggest that participating in multiple OSTEs that were specific to feedback and mentoring junior residents positively reinforces the core interpersonal and communication skills discussed in the didactic portion and practiced in the interactive portions of the program.

The data gathered from post-program evaluation suggests the program achieved success at three different levels of Kirkpatrick’s evaluation schema [8]. Participants had a positive reaction to the experience (level 1) and learned critical leadership skills (level 2) that will help them be successful in the role of CR. Seventy-two percent of the residents stated they would recommend a similar experience to colleagues, 87% stated the program was useful, and 92% agreed that the program met their learning needs, while 90% of the residents agreed or strongly agreed that the objectives were met. In addition, the post-program evaluation suggests that the skills learned will lead to behavior change and overall organization impact (level 3). Seventy-six percent felt the course would help them to be more effective at work. CRs also reported positive comments about their OSTE experience: ‘I enjoyed and felt the OSTEs were valuable for self-discovery;’ ‘The simulations were good and so was the feedback after the OSTE (by standardized learner);’ ‘Good scenarios, the practice will aid me in teaching junior residents;’ ‘The OSTEs were true to life.’ Themes identified in the feedback from CRs included the observation that self-assessment is helpful in increasing self-awareness and the opportunity to practice mentoring residents and apply the S-FED (Self Assessment; Feedback; Encouragement; Direction) coaching model is instrumental in the development of communication and feedback competencies [5]. Feedback was also gathered from the senior faculty who debriefed the OSTE experience with CRs. When asked about their impression of the effectiveness of the session, a representative faculty statement was that it was ‘an excellent session in which I got the impression that most of the incoming chiefs felt they benefited’.

## Conclusion

This communication showcases the efforts of one large academic healthcare organization with a large number of residencies and fellowships to centralize education programs for CRs. Our goal was to help CRs start their new role with appropriate education and be well prepared for challenges they are likely to encounter within their respective training programs, as well as perform at the level expected by their Program Directors and organizational GME leadership. Our primary focus on EI principles as a core competency is based on an extensive literature on leadership, which by extension supports EI as a tool for GME training initiatives. The pre-intervention EI scores of our participants reported are below the mean score that is expected for strong leaders [5]. This reinforced the need for participation in structured education, which included didactic content and, more importantly, provided an opportunity to practice skills and apply the EI principles in a favorable learning environment.

OSTEs designed specifically for CR physicians hold great promise for rapid and rigorous formative assessment of clinical teaching skills and support experiential curricular efforts. In addition they simulate the clinical environment where leadership and mentorship skills can be applied with individual residents and allow for standardized teaching and assessment [18,19]. As seen with prior studies, our study shows that OSTEs along with post-OSTE debriefing sessions can provide a platform by which learners (particularly CRs) can demonstrate competencies in areas that are often ignored, missed, or even considered ‘hidden’ [15,16]. CR education in these areas is essential as CRs are increasingly recognized as critically important teachers for their peers and medical students.

As with any educational intervention we encountered limitations. We acknowledge our study is limited to CRs within one health system, with the caveat that we have 100 chief residents across 21 hospitals in all disciplines of medicine with about 80 individual participants. Their exposure to EI training is minimal and that could be much more extensive, but the logistics of GME make this difficult earlier in their training. EI is best learned over time through consistent attention and feedback on EI principles and future follow-up sessions (‘come-back’ sessions) would reinforce skills introduced prior to beginning their CR role. Our post-OSTE debriefs were relatively unstructured; in the future we could develop a more structured format that could add to CR learning and self-reflection [15]. If resources allowed, we would also like our OSTE exposure to be more of a summative assessment. This could include expanding to eight total stations and allowing for a greater degree of feedback for participants. As well, we would consider inclusion of EI training for our standardized learners and faculty observers in order to provide additional collaborative feedback. We could also consider comparing the evaluations made by our CRs to the standardized learners and observers to account for the often poor self-assessment individuals tend to provide.

Our results do suggest that CRs, across multiple disciplines, who participated in a one-day combined didactic and experiential educational program can practice communication and professionalism behaviors that support core leadership and feedback skills. Optimizing these abilities are critical for success in their upcoming administrative and leadership roles. Most importantly the acquisition of this knowledge and skills can help assure the role of CR is a positive experience that may influence future career choices at academic health systems that include clinical, academic, and administrative roles. Towards this, we encouraged all CRs to return for optional ‘CR comeback’ educational sessions during their CR year. These sessions are focused on advanced learning topics and also include experiential simulation experiences. An opportunity to re-take the EI inventory prior to completion of their CR year, for additional self-awareness is also offered to all participants. An additional EI score six-months post-residency can provide further assessment of a sustained effect of this experiential program during the chief resident year.

## Note

This manuscript has not been published elsewhere and has not been submitted simultaneously for publication elsewhere. All authors have read and approved the final manuscript.
